# Ecological immunology: do sexual attraction and immunity trade‐off through a desaturase?

**DOI:** 10.1111/1744-7917.13379

**Published:** 2024-05-20

**Authors:** Ke Gao, Wout van der Heide, Daphne Muijderman, Sarah Nichols, Carmen Karwal, Peter Kuperus, Astrid T. Groot

**Affiliations:** ^1^ Institute for Biodiversity and Ecosystem Dynamics University of Amsterdam Amsterdam the Netherlands; ^2^ Department of Cell and Developmental Biology University of California San Diego La Jolla California USA; ^3^ Department of Neurobiology and Behavior Cornell University Ithaca New York USA; ^4^ University of Leiden Leiden the Netherlands

**Keywords:** *Chloridea virescens*, *delta‐11*‐*desaturase*, ICHH, parasite susceptibility, sex pheromone

## Abstract

Given the limited availability of resources in nature, sexual attractiveness may trade off with immunocompetence, as the immunocompetence handicap hypothesis (ICHH) posits. In invertebrates, a direct link between trade‐offs through hormonal/molecular effectors in sexual signals and immunity has not been found so far. Here, we assessed how variation in sexual signals affected parasite infection in two sex pheromone selected lines of the moth *Chloridea virescens*: an attractive line with a low ratio of 16:Ald/Z11‐16:Ald and an unattractive line with a high ratio. When infecting these lines with an apicomplexan parasite, we found that the attractive Low line was significantly more susceptible to the parasite infection than the unattractive High line. Since the ratio difference between these two lines is determined by a delta‐11‐desturase, we hypothesized that this desaturase may have a dual role, i.e., in the quality of the sexual signal as well as an involvement in immune response, comparable to testosterone in vertebrates. However, when we used CRISPR/cas9 to knockout *delta‐11‐desturase* in the attractive Low line, we found that the pheromonal phenotype did change to that of the High line, but the infection susceptibility did not. Notably, when checking the genomic location of *delta‐11‐desaturase* in the *C. virescens*, we found that *mucin* is adjacent to *delta‐11‐desaturase*. When comparing the *mucin* sequences in both lines, we found four nonsynonymous SNPs in the coding sequence, as well as intronic variation between the two lines. These differences suggest that genetic hitchhiking may explain the variation in susceptibility to parasitic infection.

## Graphical abstract

In two previously selected lines of the noctuid moth *Chloridea virescens*, we investigated whether attractive moths were more or less susceptibility to parasite infections. We found that the attractive Low line consistently exhibited a higher parasitic infection rate compared to the unattractive High line, suggesting a potential trade‐off between maintaining attractive sexual signals and susceptibility to parasites.

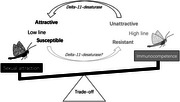

## Introduction

Sexual selection is a fundamental evolutionary process that influences the development of various ornamental traits and reproductive behaviors in animals (Andersson, 1994). These sexual traits and behaviors typically play a pivotal role in enabling individuals to evaluate and choose potential mates, and become more prevalent in a population when the benefits of these traits, i.e., an individual's reproductive success, are outweighed by their costs of survival and lifespan (Willson, 1990; Hare & Simmons, 2019). Zahavi (1975) proposed the idea that characters developing through mate preference confer handicaps on selected individuals in terms of their survival. This concept is widely known as the handicap principle, which posits that secondary sexual traits, such as the colorful tail of the male peacock, act as handicaps that are costly to produce and maintain (Zahavi, 1975; Grafen, 1990; Penn & Számadó, 2020), at least if these traits are honest signals that indicate an individual's quality or fitness (Johnstone, 1995; Roulin, 2016).

Due to limited availability of resources for animals in nature, allocating resources to the development and maintenance of sexual traits can lead to reduced resources available for other functions, such as survival (Stearns, 1989; Jacot *et al.*, 2004). One proximate mechanism, suggested by Folstad & Karter (1992), explains the costs of ornamentation. They propose that males carry exaggerated ornaments at the expense of their resistance to parasites, leading to a negative correlation between ornament and immune function. This trade‐off is often referred to as the immunocompetence handicap hypothesis (ICHH) (Folstad & Karter, 1992; Roberts *et al.*, 2004). While there is growing evidence for ICHH in vertebrates, only a handful studies have explored the ICHH hypothesis in insects, such as in crickets, beetles, flies and damselflies (Ryder & Siva‐Jothy, 2000; Rantala *et al.*, 2003b; McKean & Nunney, 2008; Cotter *et al.*, 2011; Galicia *et al.*, 2014). As far as we know, so far a direct link between trade‐offs through hormonal or molecular effectors in sexual signals and immunity has not yet been found in moths (Rantala *et al.*, 2003a, 2012; Lawniczak *et al.*, 2007; Gonzalez‐Tokman *et al.*, 2012).

The tobacco budworm *Chloridea* (*Heliothis) virescens* (Lepidoptera: Noctuidae) is a major pest in North and South America. Much is known about its reproductive biology (Groot *et al.*, 2006; Gould *et al.*, 2010; Gao *et al.*, 2020b). Females emit species‐specific sex pheromone signals to attract males. Z11‐16:Ald is their major sex pheromone component and Z9‐14:Ald is the critical secondary sex pheromone component. Previously, we found that attractiveness in *C. virescens* females is linked to the ratio of 16:Ald/Z11‐16:Ald (Groot *et al.*, 2014, 2019; van Wijk *et al.*, 2017). Females from the Low line produce the unsaturated sex pheromone components (Z11‐16:Ald and Z9‐14:Ald) in high amounts, resulting in a low ratio of 16:Ald/Z11‐16:Ald, while females from the High line produce the saturated counterparts (16:Ald and 14:Ald), resulting in a high ratio of 16:Ald/Z11‐16:Ald (Groot *et al.*, 2019). In the field, females of the Low line attracted many males, while females of the High line did not attract any males (van Wijk *et al.*, 2017). Through genetic analysis, we found that this difference is due to a stop codon in the first exon of *delta‐11‐desaturase*, resulting in a functional knockout of this desaturase (Groot *et al.*, 2019). These two lines of *C. virescens* thus form the perfect starting point to test the ICHH hypothesis in moths.

*Ophryocystis elektroscirrha* (OE) is a well‐known parasitic protozoan that infects monarch butterflies *Danaus plexippus* (McLaughlin & Myers, 1970). The transmission of the OE parasite is predominantly vertical, as females scatter spores on their eggs or host plants during oviposition, which are subsequently ingested by larvae (Altizer *et al.*, 2000). Recently, we found an OE‐like parasite on the cotton bollworm *Helicoverpa armigera*, a closely related species of *C. virescens* (Gao *et al.*, 2020a). Since larvae of this and other moth species also eat their eggshell, vertical transmission in this species likely occurs as well. Interestingly, OE‐like parasites from *H. armigera* can cross‐infect *C. virescens* in the laboratory (Gao *et al.*, 2020a). OE and OE‐like parasites infection can negatively affect host fitness, reducing eclosion, longevity, fecundity, mating success and flight ability (Bradley & Altizer, 2005; Gao *et al.*, 2020a).

In this study, we determined whether the two lines differ in their susceptibility in OE‐like infection. We also assessed the effects of OE‐like infections on sexual attraction by checking female calling behavior and sex pheromone composition. Since we consistently found that the attractive Low line was more susceptible to parasitic infections compared to the unattractive High line, we subsequently hypothesized that delta‐11‐desaturase has a pleiotropic effect in the quality of the sexual signal and in immune function. To test this hypothesis, we knocked out the *delta‐11‐desaturase* gene in the attractive Low line, using CRISPR/cas9, after which we conducted similar infection experiments. We confirmed that knocking out *delta‐11‐desaturase* changed the pheromone composition in the expected direction, but we did not find a change in susceptibility to parasite infection. In checking the genomic region of *delta‐11‐desature*, we found the *mucin* gene adjacent to *delta‐11‐desaturase*, which is known to be involved in immune responses, in which we found consistent sequence differences between the Low and High lines. These results suggest that genetic hitchhiking may be responsible for the difference in susceptibility.

## Materials and methods

### Insects

The two selection lines (named Low and High line) of *C. virescens* were generated and reared at the Institute for Biodiversity and Ecosystem Dynamics, University of Amsterdam under controlled conditions in a climate chamber (25 °C; 60% RH, 14 h light: 10 h dark) (see Groot *et al.*, 2019 for more details). Larvae were fed individually on an artificial pinto bean diet until pupation. Pupae were checked daily and newly emerged adult moths were sexed and separated into a plastic cup with 10% sucrose solution.

### OE‐like parasite infection

To acquire infected moths, we followed our previous infection protocol, as described in details in Gao *et al.* (2020a) and summarized briefly here. Third instar larvae were starved for 1–5 h, after which they were fed 1 μL of spore suspension on a piece of 1 cm^2^ artificial diet. After consumption of the diet, the larvae were transferred individually to normal diet until pupation. The spore suspension was prepared by collecting OE‐like spores from the abdomens of 1–3 infected adult moths. The spore doses were quantified by counting the number of spores in 1 μL of spore suspension under the microscope. To determine the effect of OE‐like infection in the Low and High lines, we conducted infection experiments with multiple spore doses, as follows: 13/μL, Low line: *n* = 48, High line: *n* = 46; 55/μL, Low line: *n* = 45, High line: *n* = 53; 122/μL, Low line: *n* = 27, High line: *n* = 30; 144/μL, Low line: *n* = 100, High line: *n* = 80; 153/μL, Low line: *n* = 26, High line: *n* = 28. Upon eclosion, the abdomen of adults was sampled, using a 2.5 cm diameter transparent tape, which was checked for OE‐like parasite spores under the microscope.

### Female calling behavior

To evaluate the effect of OE‐like infection on female calling behavior, virgin females from Low and High line were observed over five consecutive nights. Upon eclosion, all adult moths were sexed, and placed individually into transparent plastic beakers (473 mL) covered with nylon gauze. Based on checking infection status, females were categorically separated into three groups: control (moths that were never treated with OE‐like spores), treated (moths that had been treated with OE‐like spores as larvae, but the adults were not infected) and infected (moths that had been treated with OE‐like spores as larvae and the adults were infected). All the moths were supplied with 10% sucrose solution. Female calling behavior was observed under red light at 30 min intervals throughout scotophase. Observations were conducted in the same climatic conditions in which the insects were reared.

### Female pheromone analysis

To determine the effect of OE‐like infection on female sex pheromone composition, the glands of 5‐d‐old females from Low and High line in control (Low line, *n* = 26; High line, *n* = 31), treated (Low line, *n* = 31; High line, *n* = 35) and infected (Low line, *n* = 16; High line, *n* = 10) groups were dissected during the peak time of calling during scotophase. The details of extraction have been described in Groot *et al.* (2010), and summarized here: pheromone glands were immersed approximately 30 min in conical vials containing a solution of 50 μL hexane and 200 ng of pentadecane as internal standard. All pheromone samples were analyzed in a HP7890 Gas Chromatograph (GC) with a 7683 automatic injector. The peaks of sex pheromone were identified and integrated manually compared their retention time to a synthetic pheromone blend of *C. virescens*.

### Creating a *C. virescens* lab population homozygous for the attractive Low line allele

To determine whether *delta‐11‐desaturase* is responsible for differences in susceptibility for OE‐like infection, we knocked out this gene in the attractive Low line. To ensure that the High allele with the stop codon in *delta‐11‐desaturase* was absent, we first established a lab population homozygous for the attractive Low line allele as follows. DNA was extracted from complete adult legs by adding 100 μL of stirred 10% Chelex (Sigma‐Aldrich Chelex) and 5 μL of proteinase K (Thermo scientific). 8 μL of PCR mastermix was added for every 2 μL of template DNA. The mastermix consisted of 4.82 μL of Milli‐Q purified water, 1 μL of 10× DreamTaq buffer (Thermo Scientific), 1 μL of dNTPs, 0.5 μL of BSA (New England Biolabs), 0.3 μmol/L of each primer (D11 exon 3F & D11 exon 4R; see Table S2), and 0.08 μL of DreamTaq DNA‐polymerase (5 U/μL; Thermo Scientific). DNA was amplified through PCR in a BioRadT100TM Thermal Cycler following the program: 94 °C for 2 min, 35 cycles (45 s at 94 °C, 45 s at 57.3 °C, 1 min at 72 °C). The PCR products and the genotypes were then analyzed through 1.5 % agarose gel. A total of 24 pairs of homozygous Low line moths were established and used to setup for CRISPR injection.

### Knockout of *delta‐11‐desaturase* in the Low line through CRISPR‐cas9

To knock out *delta‐11‐desaturase*, we used CRISPR‐cas9 as follows. Three RNA guides were designed against exon three of the *delta‐11‐desaturase*, using the Intergrated DNA Technologies (IDT) custom Alt‐R CRISPR‐Cas9 gRNA design tool and the Low line genomic DNA sequence as a target (see Table S2). Lyophilized tracrRNA and crRNAs were dissolved in a nuclease free buffer in a 1 : 1 ratio. An annealing step was then carried out at 94 °C for 2 min, after which the crRNA:tracrRNA duplex was left to cool for 2 min at room temperature. Aliquots of sgRNAs targeting the *delta‐11‐desaturase* were mixed with Cas9 and an HvSC‐BF (or ‘scarlet’) reporter sgRNA. The final solution for embryo injection consisted of four sgRNAs (200 pmol) and IDT Alt‐R Cas9 (100 pmol).

Embryo injection occurred between 0.5 and 1 h after oviposition using a FemtoJet (Eppendorf) injector. Harvard Apparatus 1.0 mm (OD) capillaries were used for needle pulling. A total of 1271 freshly laid eggs were collected and injected, from which only 24 larvae survived, which was likely due to the inexperience of the egg injector. These larvae were reared to adulthood and genotyped. To screen the mutant individuals, we took one foreleg of newly hatched adults for DNA extraction and genotyping, as described above. PCR amplification followed the program: 94 °C for 2 min, 35 cycles (45 s at 94 °C, 45 s at 56.8 °C, 1 min at 72 °C) with a pair of specific primers: scrn1 (see Table S2). PCR products were assessed on low‐melting agarose gels. From the 11 G0 individuals that were sequenced by Macrogen EZ‐seq (Amsterdam, Netherlands), one individual carried the mutation, with an 18 bp deletion and two 1 bp insertions.

To obtain a homozygous CRISPR line, the G0 mutant adult was crossed to wild type homozygous Low line adults. G1 heterozygote males and females were crossed to obtain G2 homozygote mutants. The G1 and G2 adults were genotyped as described above, whereby the leg was collected as soon as the moths emerged. The offspring of the homozygous and heterozygous G2 females were used for the OE‐like infection experiment.

### Phenotyping the CRISPR lines

To determine whether the knockout of *delta‐11‐desaturase* was successful, we analyzed the sex pheromone composition in the G2 CRISPRed females, as described above. Pheromone glands were extracted from 3‐ to 4‐d‐old virgin, homozygous CRISPR females (*n* = 25) and heterozygous CRISPR females (*n* = 21). To determine the effect of OE‐like parasite on the CRISPRed moths, we conducted OE‐like infection experiments as described above. This time we used a spore solution of 113 spores/μL and larvae of Low line (*n* = 49), High line (*n* = 18), CRISPR homozygotes (*n* = 20) and CRISPR heterozygotes (*n* = 39) were infected at the same time to be able to compare all different lines.

### Genomic sequence variation adjacent to *delta‐11‐desaturase*

As the knockout of *delta‐11‐desaturase* did not result in lower susceptibility of OE‐like infections, we compared the genomic sequences between the Low and High lines adjacent to *delta‐11‐desature*. As we found *mucin* in close vicinity to *delta‐11‐desaturase*, and this gene is known to have anti‐pathogenic effects/involved in immune responses (Syed *et al.*, 2008; Shangguan *et al.*, 2018), our first analysis was focused on assessing sequence variation in this gene. For this analysis, we extracted genomic DNA from Low and High line individuals, using the CTAB protocol as described in Gao *et al.* (2020a). Primers were designed using Primer3 software. To sequence the *mucin* gene, four pairs of primers were used (see Table S2), and PCR amplification were as follows: 98 °C for 30 s, 34 cycles (20 s at 98 °C, 20 s at 61 °C, 260 s at 72 °C). The PCR products were examined on a 1.5% agarose gel. The sequences of 7 Low and 6 High individuals were checked and aligned using CodonCode Aligner. The 3D structure of the *mucin* protein was run in AlphaFold software.

### Data analysis

All statistical analyses were performed in R, version 4.0.2 (2020). The difference of OE‐like infection rate between Low and High line was compared, using PROP test. Differences in percentages of females calling over five consecutive nights between the control, infected, and treated groups were analyzed using Fisher's exact test. To evaluate the effect of OE‐like infection on female sex pheromone in Low and High line, the relative amounts of the compounds in the pheromone blend between control, infected and treated females were compared by a multivariate analysis of variance (MANOVA), while the total amounts of pheromone and each compound between control, infected and treated females was compared with a one‐way ANOVA, followed by Tukey‐Kramer HSD test for multiple comparisons (package: multcomp). The ratio of 16:Ald and Z11‐16:Ald in the female pheromone between Low line, and High line, CRISPR homozygote and CRISPR heterozygote was log10(*x* + 1)‐transformed to normalize the data, and then compared with one‐way ANOVA, followed by Tukey‐Kramer HSD test for multiple comparisons. Differences in OE‐like infection between Low line, High line, CRISPR heterozygote and CRISPR homozygote moths was analyzed using chi‐square tests.

## Results

### OE‐like infection rate

**Fig. 1:**
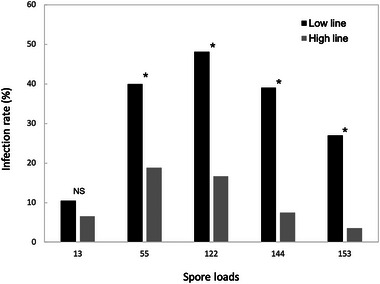
Infection rate of OE‐like parasite between Low and High line of *C. virescens*. Total number of larvae treated with OE‐like spores (13/μL, Low line: *n* = 48, High line: *n* = 46; 55/μL, Low line: *n* = 45, High line: *n* = 53; 122/μL, Low line: *n* = 27, High line: *n* = 30; 144/μL, Low line: *n* = 100, High line: *n* = 80; 153/μL, Low line: *n* = 26, High line: *n* = 28). Significant differences are indicated by asterisks (*P* < 0.05); NS: not significant.

The attractive Low line of *C. virescens* showed a consistently higher infection rate compared to the unattractive High line (spore does: 55/μL, *χ^2^* = 4.34, df = 1, *P* = 0.037; 122/μL, *χ^2^* = 5.14, df = 1, *P* = 0.023; 144/μL, *χ^2^* = 21.87, df = 1, *P* < 0.001; 153/μL, *χ^2^* = 4.12, df = 1, *P* = 0.042), except when treated with low spore does (13/μL, *χ^2^* = 0.094, df = 1, *P* = 0.759) (Fig. 1).

### Effect of OE‐like infection on female calling behavior

**Fig. 2:**
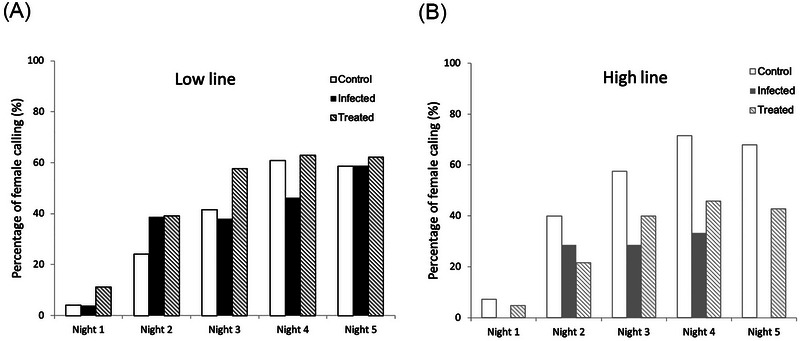
Calling behavior of virgin *C. virescens* females throughout the scotophase over five consecutive nights in control, infected and treated groups, in (A) Low line females; (B) High line females. The number of females observed over five consecutive nights is given in Table S1.

In both Low and High line, OE‐like infection did not affect the percentage of females calling over five consecutive nights in control, infected and treated groups (Fig. 2). However, in the treated group, Low line females called significantly more on the second night compared to the High line (Fisher's exact test, *P* = 0.036), and close to significantly more on the third (Fisher's exact test, *P* = 0.055) and forth night (Fisher's exact test, *P* = 0.069). Female calling was not significantly different on the first (Fisher's exact test, *P* = 0.206) or fifth night (Fisher's exact test, *P* = 0.14) (Fig. S1).

### Effect of OE‐like infection on female pheromone signal

**Fig. 3:**
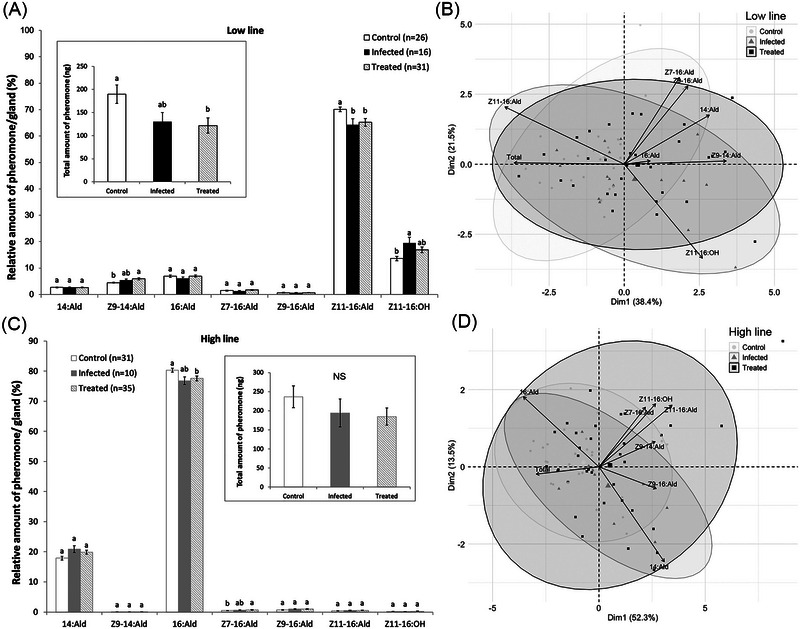
Pheromone composition of *C. virescens* females within control, infected and treated group. Relative amounts of pheromone (A) in the Low line, (C) in the High line. Inserts: total amount of pheromone. Principal component analysis (PCA) of female pheromone blends (B) in the Low line, (D) in the High line. Significant differences are indicated by different letters (*P* < 0.05); NS: not significant.

In the attractive Low line, OE‐like infections affected the relative amount of the major pheromone component Z11‐16:Ald, which was significantly lower in treated (Tukey's post hoc test: *P* = 0.035) and infected females (Tukey's post hoc test: *P* = 0.038) compared to control females. The amount of Z9‐14:Ald was higher in treated females than in control females (Tukey's post hoc test: *P* = 0.008). The relative amount of Z11‐16:OH was higher in infected females than in control females (Tukey's post hoc test: *P* = 0.011). In addition, the total amount of pheromone was significantly lower in treated females compared to control females (Tukey's post hoc test: *P* = 0.024) (Fig. 3A).

In the unattractive High line, OE‐like infections affected the relative amount of the major pheromone compound 16:Ald, which was significantly lower in treated females compared to control females (Tukey's post hoc test: *P* = 0.031), while the amount of Z7‐16:Ald was higher in treated females than in control females (Tukey's post hoc test: *P* = 0.038). However, the total amount of pheromone in the Low line did not differ between control, treated and infected females (ANOVA, *P* = 0.34) (Fig. 3C).

### Knocking out the *delta11‐desaturase* gene in Low line

**Fig. 4:**
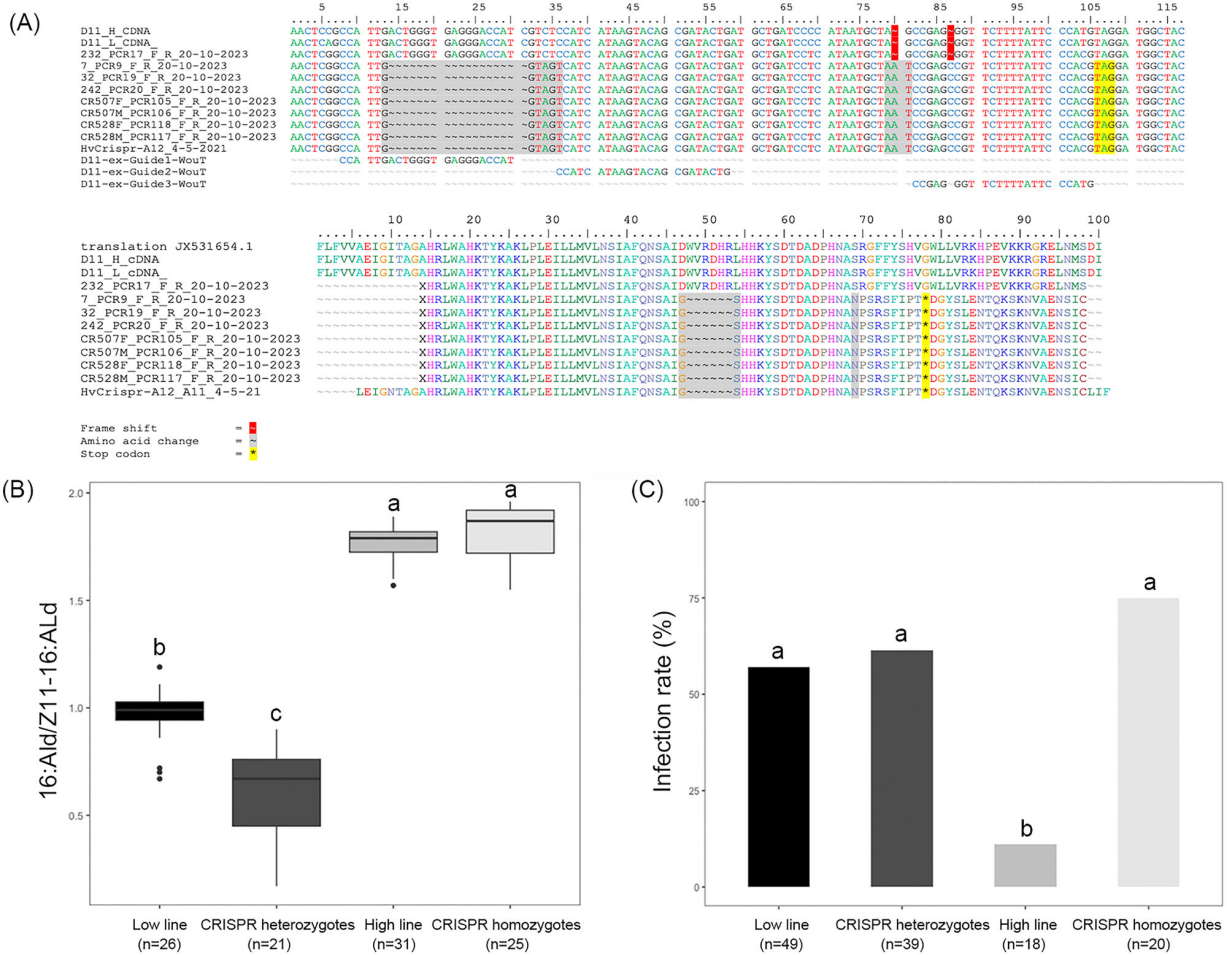
(A) Sequence of the CRISPR/Cas9 induced mutation in the Low line *C. virescens delta11‐desaturase* gene. Sequence modifications are highlighted in red (frame shift) and yellow (stop codon), resulting amino acid changes are shown in gray. (B) Female pheromone ratio of 16:Ald to Z11‐16:Ald and (C) OE‐like infection rates (%) in Low line, CRISPR heterozygous, High line, and CRISPR‐homozygous moths.

When we examined the sex pheromone composition in the CRISPRed females, we found that the CRISPR homozygote females had a significantly higher ratio of 16:Ald/Z11‐16:Ald compared to the CRISPR heterozygote females, as expected (Tukey's post hoc test: *P* < 0.001). The ratio of 16:Ald/Z11‐16:Ald of the homozygous CRISPR females was similar to the ratio in the unattractive High line (Tukey's post hoc test: *P* = 0.409), while the ratio of 16:Ald/Z11‐16:Ald in the heterozygous CRISPR females was significantly lower than the ratio of the attractive Low line (Tukey's post hoc test: *P* < 0.001) (Fig. 4).

When assessing the OE‐like infection, we found that both the CRISPR homozygotes (75%) and the CRISPR heterozygotes (61.5%) exhibited a similarly high infection rate, comparable to that of the attractive Low line (57.1%), in contrast to the lower infection rate observed in the unattractive High line (11.1%) (Fig. 4)

### Differences in the *mucin* gene

**Fig. 5:**
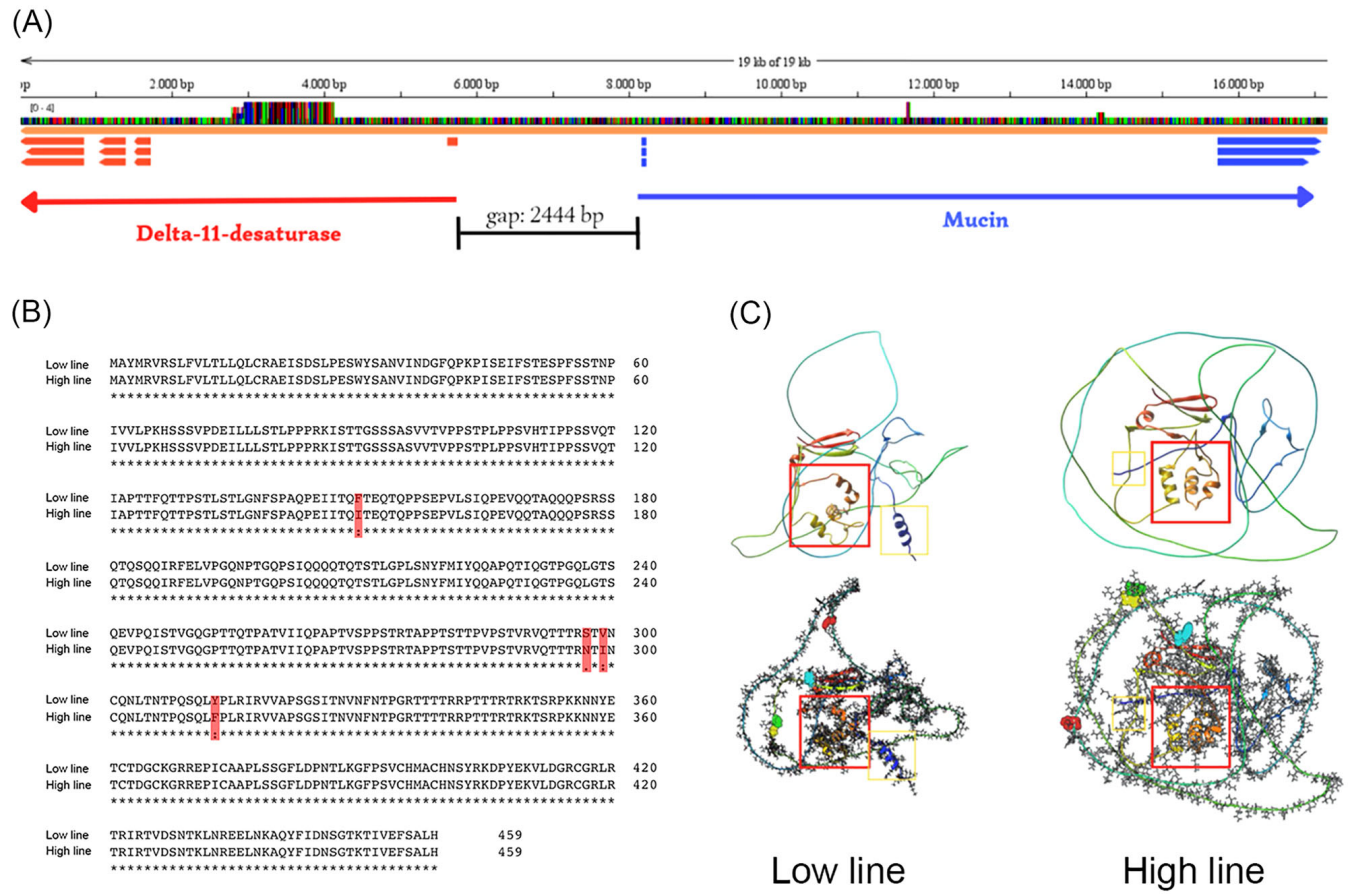
(A) A‐lignment of *delta‐11‐desaturase* (red) and *mucin* (blue). (B) Mucin amino acid sequences between Low and High line. (C) Predicted 3D structures of mucin in Low and High line.

In checking the *mucin* gene adjacent to *delta‐11‐desaturase*, we found that this gene consists of three exons and two introns, with a coding sequence (cds) of 1380 bp and 460 amino acids long. When comparing the amino acid sequences of *mucin* between Low and High line, within the cds we found four consistent nonsynonymous amino acid mutations at codons 150, 297, 299, and 313, which were located in the second exon (see Fig. 5A, B). Additionally, we found sequence length variation in the first intron between the Low and High line sequences, with the Low line having an intron of 6235 bp and the High line having an intron of 10 023 bp. When predicting the 3D structures of *mucin* using AlphaFold, we found differences in protein structure between Low and High line, as highlighted in Fig. 5C.

## Discussion

Here we examined how parasitic infection affected the sexual signals and behaviors, as well as susceptibility to infection between two different selected lines of *C. virescens* that differ in sexual attraction, which is due to a mutation in *delta‐11‐desaturase* (Groot *et al.*, 2019). We found that the attractive Low line consistently showed a higher OE‐like infection rate compared to the unattractive High line. We also found that OE‐like infections had different effects on the calling behavior, sex pheromone, or immunocompetence in the attractive and unattractive lines. Below we discuss our results in relation to trade‐offs between sexual attractiveness and parasite susceptibility.

Since we found that attractive Low line moths were consistently more susceptible to parasitic infection than unattractive High line moths, our results imply a trade‐off between maintaining and expressing an attractive sexual signals and parasite susceptibility. This finding is in line with the prediction of the ICHH hypothesis that states that investment in reproductive traits, such as sexual attraction, trades off with resistance to parasitic challenges. Empirical evidence supporting the ICHH hypothesis has been found before in other insects, including crickets, beetles, flies, and damselflies (Ryder & Siva‐Jothy, 2000; Rantala *et al.*, 2003a; McKean & Nunney, 2008; Cotter *et al.*, 2011; Gonzalez‐Tokman *et al.*, 2012; Galicia *et al.*, 2014), but so far not in Lepidoptera, as far as we are aware.

In vertebrates, there is more supporting evidence for the ICHH hypothesis, and testosterone has been found to play a dual role by contributing to the development of secondary sexual traits, but also suppressing immune function (Folstad & Karter, 1992; Hasselquist *et al.*, 1999). In invertebrates, a few studies have suggested that juvenile hormone may serve roles analogous to testosterone in vertebrates, affecting resource allocation for sexual traits and immune function (Rantala *et al.*, 2003a; Flatt *et al.*, 2005; Gilbert *et al.*, 2016). For instance, when injecting male mealworm beetles *Tenebrio molitor* with juvenile hormone, this resulted in an increase in the attractiveness of male pheromones while concomitantly suppressing encapsulation and phenoloxidase activity (Rantala *et al.*, 2003a).

Since we previously found significant differences in sex pheromone composition within *C. virescens* that could be selected for, resulting in an attractive Low line and an unattractive High line without any other apparent fitness effects (Groot *et al.*, 2014, 2019), we were in the unique position to compare parasite susceptibility between these two lines. Although investment of sexual signals helps individuals attract mates and increase the chances of reproductive success, the costs of sexual signals can be significant. Several studies have shown that developing and maintaining attractive sexual signals can negatively affect overall fitness by reducing their longevity (e.g., time and energy of foraging) or survival rate (e.g., more vulnerable to physical damages or injuries, and predators or parasites) (Olson & Owens, 1998; Scharf *et al.*, 2013). In moths, several studies suggest that sex pheromone production and/or emission can be costly in terms of quality and quantity (Harari *et al.*, 2011; Foster & Anderson, 2015; Gao *et al.*, 2021). As we found that the attractive Low line consistently showed a higher OE‐like infection rate compared to the unattractive High line, our results also indicate that there is a trade‐off between sexual attraction and immune function.

In this study, we found that OE‐like parasite infections did not affect the calling behavior or the sex pheromone composition in both the attractive Low line and the unattractive High line, compared to treated or control females. However, we would like to point out that our experiments were conducted under ideal laboratory conditions, where optimal food resources were supplied for both larvae and adults, which might compensate for the parasitic challenge. Several studies have shown the benefits of optimal nutrition for immune function for insects (Cotter *et al.*, 2011; Singer *et al.*, 2014; Henry *et al.*, 2018; Ponton *et al.*, 2023), for example, fruit flies *Drosophila melanogaster* raised under nutrient‐rich diet exhibited enhanced resistance to bacterial infections compared to those reared under resource‐limited conditions (Unckless *et al.*, 2015). In nature, it is more likely that limited resources and suboptimal conditions are available (Schmid‐Hempel, 2005), which may amplify the effects of infections (Cotter & Al Shareefi, 2022).

As we previously found a stop codon in *delta‐11‐desaturase* to underlie the sex pheromone differences (Groot *et al.*, 2019), we tested whether this gene could play a dual role by knocking out this gene using CRISPR/Cas9. In our loss‐of‐function studies, we did find that knocking out *delta‐11‐desaturase* in the attractive Low line converted the sex pheromone to that of the unattractive High line, but we did not find a change in parasite susceptibility. Thus, *delta‐11‐desaturase* does not appear to play a role in immunocompetence.

Interestingly, however, we found *mucin* to be adjacent to *delta‐11‐desaturase* in the *C. virescens* genome. Mucins, as glycoproteins, are involved in a range of biological functions, including immune defense, where they play a pivotal role in pathogen recognition and immune system activation (Syed *et al.*, 2008; Shangguan *et al.*, 2018). When comparing the sequence of this gene between the Low and High line, we identified four nonsynonymous amino acid substitutions within the second exon, as well as intron‐size differences. The genetic variations we observed in the *mucin* gene between the Low and High lines suggest that the difference in parasite susceptibility between the attractive Low and the unattractive High line may be due to genetic hitchhiking (Hedrick, 1982; Barton, 2000). Possibly, the close distance between *mucin* and *delta‐11‐desaturase* resulted in the consistent differences in the parasite susceptibility that we observed between the Low and High line.

In conclusion, our study provides empirical support for the ICHH hypothesis, as we found a trade‐off between sexual attractiveness and parasite susceptibility. The mechanism underlying this trade‐off is not clear yet, but we can reject the hypothesis that *delta‐11‐desaturase* has a dual function in sex pheromone production and parasite resistance. Whether and how the genomically adjacent *mucin* is involved in parasite resistance remains to be further investigated.

## Disclosure

The authors declare that there is no conflict of interest.

## Supporting information


**Table S1** The number of females calling in the Low and High line observed over five consecutive nights.
**Table S2** Primers and sgRNA guides used in this study.
**Fig. S1** Comparing the females calling behavior in the treated group between Low and High line throughout the scotophase over five consecutive nights.

## References

[ins13379-bib-0001] Altizer, S.M. , Oberhauser, K.S. and Brower, L.P. (2000) Associations between host migration and the prevalence of a protozoan parasite in natural populations of adult monarch butterflies. Ecological Entomology, 25, 125–139.

[ins13379-bib-0002] Andersson, M. (1994) Sexual Selection. Princeton University Press.

[ins13379-bib-0003] Barton, N.H. (2000) Genetic hitchhiking. Philosophical Transactions of the Royal Society of London. Series B: Biological Sciences, 355, 1553–1562. 11127900 10.1098/rstb.2000.0716 PMC1692896

[ins13379-bib-0004] Bradley, C.A. and Altizer, S. (2005) Parasites hinder monarch butterfly flight: implications for disease spread in migratory hosts. Ecology Letters, 8, 290–300.

[ins13379-bib-0005] Cotter, S.C. and Shareefi, E.A. (2022) Nutritional ecology, infection and immune defence—exploring the mechanisms. Current Opinion in Insect Science, 50, 100862. 34952240 10.1016/j.cois.2021.12.002

[ins13379-bib-0006] Cotter, S.C. , Simpson, S.J. , Raubenheimer, D. and Wilson, K. (2011) Macronutrient balance mediates trade‐offs between immune function and life history traits. Functional Ecology, 25, 186–198.

[ins13379-bib-0007] Flatt, T. , Tu, M. and Tatar, M. (2005) Hormonal pleiotropy and the juvenile hormone regulation of *Drosophila* development and life history. BioEssays, 27, 999–1010. 16163709 10.1002/bies.20290

[ins13379-bib-0008] Folstad, I. and Karter, A.J. (1992) Parasites, bright males, and the immunocompetence handicap. The American Naturalist, 139, 603–622.

[ins13379-bib-0009] Foster, S.P. and Anderson, K.G. (2015) Sex pheromones in mate assessment: analysis of nutrient cost of sex pheromone production by females of the moth *Heliothis virescens* . The Journal of Experimental Biology, 218, 1252–1258. 25722008 10.1242/jeb.119883

[ins13379-bib-0010] Galicia, A. , Cueva del Castillo, R. and Contreras‐Garduño, J. (2014) Is sexual dimorphism in the immune response of *Gryllodes sigillatus* related to the quality of diet? International Scholarly Research Notices, 2014, 329736.

[ins13379-bib-0011] Gao, K. , Muijderman, D. , Nichols, S. , Heckel, D.G. , Wang, P. , Zalucki, M.P. *et al.* (2020a) Parasite‐host specificity: a cross‐infection study of the parasite *Ophryocystis elektroscirrha* . Journal of Invertebrate Pathology, 170, 107328. 31952966 10.1016/j.jip.2020.107328

[ins13379-bib-0012] Gao, K. , Wijk, M.v. , Clement, Z. , Egas, M. and Groot, A.T. (2020b) A life‐history perspective on sexual selection in a polygamous species. BMC Evolutionary Biology, 20, 1–10. 32380947 10.1186/s12862-020-01618-3 PMC7206733

[ins13379-bib-0013] Gao, K. , Wijk, M.V. , Dang, Q.T.D. , Heckel, D.G. , Zalucki, M.P. and Groot, A.T. (2021) How healthy is your mate ? Sex‐specific consequences of parasite infections in the moth *Helicoverpa armigera* . Animal Behaviour, 178, 105–113.

[ins13379-bib-0014] Gilbert, R. , Karp, R.D. and Uetz, G.W. (2016) Effects of juvenile infection on adult immunity and secondary sexual characters in a wolf spider. Behavioral Ecology, 27, 946–954.

[ins13379-bib-0015] Gonzalez‐Tokman, D.M. , Munguía‐Steyer, R. , Gonzalez‐Santoyo, I. , Baena‐Díaz, F.S. and Cordoba‐Aguilar, A. (2012) Support for the immunocompetence handicap hypothesis in the wild: hormonal manipulation decreases survival in sick damselflies. Evolution, 66, 3294–3301. 23025617 10.1111/j.1558-5646.2012.01678.x

[ins13379-bib-0016] Gould, F. , Estock, M. , Hillier, N.K. , Powell, B. , Groot, A.T. , Ward, C.M. *et al.* (2010) Sexual isolation of male moths explained by a single pheromone response QTL containing four receptor genes. Proceedings of the National Academy of Sciences USA, 107, 8660–8665. 10.1073/pnas.0910945107 PMC2889340 20404144

[ins13379-bib-0017] Grafen, A. (1990) Biological signals as handicaps. Journal of Theoretical Biology, 144, 517–546. 2402153 10.1016/s0022-5193(05)80088-8

[ins13379-bib-0018] Groot, A.T. , Claßen, A. , Staudacher, H. , Schal, C. and Heckel, D.G. (2010) Phenotypic plasticity in sexual communication signal of a noctuid moth. Journal of Evolutionary Biology, 23, 2731–2738. 21121086 10.1111/j.1420-9101.2010.02124.x

[ins13379-bib-0019] Groot, A.T. , Horovitz, J.L. , Hamilton, J. , Santangelo, R.G. , Schal, C. and Gould, F. (2006) Experimental evidence for interspecific directional selection on moth pheromone communication. Proceedings of the National Academy of Sciences USA, 103, 5858–5863. 10.1073/pnas.0508609103 PMC1458663 16585529

[ins13379-bib-0020] Groot, A.T. , Schöfl, G. , Inglis, O. , Donnerhacke, S. , Classen, A. , Schmalz, A. *et al.* (2014) Within‐population variability in a moth sex pheromone blend: genetic basis and behavioural consequences. Proceedings of the Royal Society B: Biological Sciences, 281, 20133054. 10.1098/rspb.2013.3054 PMC3924083 24500170

[ins13379-bib-0021] Groot, A.T. , Wijk, M.v. , Villacis‐Perez, E. , Kuperus, P. , Schöfl, G. , Veldhuizen, D.v. *et al.* (2019) Within‐population variability in a moth sex pheromone blend, part 2: selection towards fixation. Royal Society Open Science, 6, 182050. 31032049 10.1098/rsos.182050 PMC6458377

[ins13379-bib-0022] Harari, A.R. , Zahavi, T. and Thiéry, D. (2011) Fitness cost of pheromone production in signaling female moths. Evolution, 65, 1572–1582. 21644949 10.1111/j.1558-5646.2011.01252.x

[ins13379-bib-0023] Hare, R.M. and Simmons, L.W. (2019) Sexual selection and its evolutionary consequences in female animals. Biological Reviews, 94, 929–956. 30484943 10.1111/brv.12484

[ins13379-bib-0024] Hasselquist, D. , Marsh, J.A. , Sherman, P.W. and Wingfield, J.C. (1999) Is avian humoral immunocompetence suppressed by testosterone? Behavioral Ecology and Sociobiology, 45, 167–175.

[ins13379-bib-0025] Hedrick, P.W. (1982) Genetic hitchhiking: a new factor in evolution? Bioscience, 32, 845–853.

[ins13379-bib-0026] Henry, M.A. , Gasco, L. , Chatzifotis, S. and Piccolo, G. (2018) Does dietary insect meal affect the fish immune system? The case of mealworm, *Tenebrio molitor* on European sea bass, *Dicentrarchus labrax* . Developmental & Comparative Immunology, 81, 204–209. 29229441 10.1016/j.dci.2017.12.002

[ins13379-bib-0027] Jacot, A. , Scheuber, H. and Brinkhof, M.W.G. (2004) Costs of an induced immune response on sexual display and longevity in field crickets. Evolution, 58, 2280–2286. 15562690 10.1111/j.0014-3820.2004.tb01603.x

[ins13379-bib-0028] Johnstone, R.A. (1995) Sexual selection, honest advertisement and the handicap principle: reviewing the evidence. Biological Reviews, 70, 1–65. 7718697 10.1111/j.1469-185x.1995.tb01439.x

[ins13379-bib-0029] Lawniczak, M.K.N. , Barnes, A.I. , Linklater, J.R. , Boone, J.M. , Wigby, S. and Chapman, T. (2007) Mating and immunity in invertebrates. Trends in Ecology and Evolution, 22, 48–55. 17028056 10.1016/j.tree.2006.09.012

[ins13379-bib-0030] McKean, K.A. and Nunney, L. (2008) Sexual selection and immune function in *Drosophila melanogaster* . Evolution, 62, 386–400. 18070086 10.1111/j.1558-5646.2007.00286.x

[ins13379-bib-0031] McLaughlin, R.E. and Myers . (1970) *Ophryocystis elektroscirrha* sp. n., a neogregarine pathogen of the monarch butterfly *Danaus plexippus* (L.) and the Florida queen butterfly *D. gilippus berenice* Cramer. The Journal of Protozoology, 17, 300–305.

[ins13379-bib-0032] Olson, V.A. and Owens, I.P.F. (1998) Costly sexual signals: are carotenoids rare, risky or required? Trends in Ecology & Evolution, 13, 510–514. 21238418 10.1016/s0169-5347(98)01484-0

[ins13379-bib-0033] Penn, D.J. and Számadó, S. (2020) The Handicap Principle: how an erroneous hypothesis became a scientific principle. Biological Reviews, 95, 267–290. 31642592 10.1111/brv.12563 PMC7004190

[ins13379-bib-0034] Ponton, F. , Tan, Y.X. , Forster, C.C. , Austin, A.J. , English, S. , Cotter, S.C. *et al.* (2023) The complex interactions between nutrition, immunity and infection in insects. Journal of Experimental Biology, 226, jeb245714. 38095228 10.1242/jeb.245714

[ins13379-bib-0035] Rantala, M.J. , Kortet, R. , Kotiaho, J.S. , Vainikka, A. and Suhonen, J. (2003a) Condition dependence of pheromones and immune function in the grain beetle *Tenebrio molitor* . Functional Ecology, 17, 534–540.

[ins13379-bib-0036] Rantala, M.J. , Moore, F.R. , Skrinda, I. , Krama, T. , Kivleniece, I. , Kecko, S. *et al.* (2012) Evidence for the stress‐linked immunocompetence handicap hypothesis in humans. Nature Communications, 3, 694. 10.1038/ncomms1696 PMC4355638 22353724

[ins13379-bib-0037] Rantala, M.J. , Vainikka, A. and Kortet, R. (2003b) The role of juvenile hormone in immune function and pheromone production trade‐offs: a test of the immunocompetence handicap principle. Proceedings of the Royal Society of London. Series B: Biological Sciences, 270, 2257–2261. 10.1098/rspb.2003.2472 PMC1691508 14613612

[ins13379-bib-0038] Roberts, M.L. , Buchanan, K.L. and Evans, M.R. (2004) Testing the immunocompetence handicap hypothesis: a review of the evidence. Animal Behaviour, 68, 227–239.

[ins13379-bib-0039] Roulin, A. (2016) Condition‐dependence, pleiotropy and the handicap principle of sexual selection in melanin‐based colouration. Biological Reviews, 91, 328–348. 25631160 10.1111/brv.12171

[ins13379-bib-0040] Ryder, J.J. and Siva‐Jothy, M.T. (2000) Male calling song provides a reliable signal of immune function in a cricket. Proceedings of the Royal Society of London. Series B: Biological Sciences, 267, 1171–1175. 10.1098/rspb.2000.1125 PMC1690662 10902682

[ins13379-bib-0041] Scharf, I. , Peter, F. and Martin, O.Y. (2013) Reproductive trade‐offs and direct costs for males in arthropods. Evolutionary Biology, 40, 169–184.

[ins13379-bib-0042] Schmid‐Hempel, P. (2005) Evolutionary ecology of insect immune defenses. Annual Review of Entomology, 50, 529–551. 10.1146/annurev.ento.50.071803.130420 15471530

[ins13379-bib-0043] Shangguan, X. , Zhang, J. , Liu, B. , Zhao, Y. , Wang, H. , Wang, Z. *et al.* (2018) A mucin‐like protein of planthopper is required for feeding and induces immunity response in plants. Plant Physiology, 176, 552–565. 29133370 10.1104/pp.17.00755 PMC5761773

[ins13379-bib-0044] Singer, M.S. , Mason, P.A. and Smilanich, A.M. (2014) Ecological immunology mediated by diet in herbivorous insects. Integrative and Comparative Biology, 54, 913–921. 24951503 10.1093/icb/icu089

[ins13379-bib-0045] Stearns, S.C. (1989) Trade‐offs in life‐history evolution. Functional Ecology, 3, 259–268.

[ins13379-bib-0046] Syed, Z.A. , Härd, T. , Uv, A. and Dijk‐Härd, I.F.v. (2008) A potential role for *Drosophila* mucins in development and physiology. PLoS ONE, 3, e3041. 18725942 10.1371/journal.pone.0003041 PMC2515642

[ins13379-bib-0047] Unckless, R.L. , Rottschaefer, S.M. and Lazzaro, B.P. (2015) The complex contributions of genetics and nutrition to immunity in *Drosophila melanogaster* . PLoS Genetics, 11, e1005030. 25764027 10.1371/journal.pgen.1005030 PMC4357385

[ins13379-bib-0048] Wijk, M.v. , Heath, J. , Lievers, R. , Schal, C. and Groot, A.T. (2017) Proximity of signallers can maintain sexual signal variation under stabilizing selection. Scientific Reports, 7, 18101. 29273813 10.1038/s41598-017-17327-9 PMC5741759

[ins13379-bib-0049] Willson, M.F. (1990) Sexual selection in plants and animals. Trends in Ecology & Evolution, 5, 210–214. 21232357 10.1016/0169-5347(90)90133-X

[ins13379-bib-0050] Zahavi, A. (1975) Mate selection‐a selection for a handicap. Journal of Theoretical Biology, 53, 205–214. 1195756 10.1016/0022-5193(75)90111-3

