# Supplementary material for: Ecological immunology: do sexual attraction and immunity trade‐off through a desaturase?
Source: Insect Sci. 2024 May 20;32(1):290–300. doi: 10.1111/1744-7917.13379 (PMC11824886; doi:10.1111/1744-7917.13379)
Supplement: Supplementary file 1 — Table S1 The number of females calling in the Low and High line observed over five consecutive nights. Table S2 Primers and sgRNA guides used in this study. Fig. S1 Comparing the females calling behavior in the treated group between Low and High line throughout the scotophase over five consecutive nights. [file INS-32-290-s001.docx]

Table S1 The number of females calling in the Low and High line observed over five consecutive nights.

| Line | Treatment | Night1 | Night2 | Night3 | Night4 | Night5 |
| --- | --- | --- | --- | --- | --- | --- |
| Low line | Control | 50 | 54 | 53 | 51 | 41 |
|  | Infected | 26 | 31 | 29 | 26 | 17 |
|  | Treated | 63 | 64 | 66 | 62 | 37 |
| High line | Control | 41 | 45 | 47 | 42 | 28 |
|  | Infected | 7 | 7 | 7 | 6 | 0 |
|  | Treated | 64 | 65 | 65 | 59 | 28 |

Table S2 Primers and sgRNA guides used in this study

| 1. Genotyping the allele of the two lines primers | |
| --- | --- |
| Name | Sequences |
| D11 exon 3F | GCAGAAATAGGGATCACGGC |
| D11 exon 4R | CGTTTACGCACCACTTCACT |
| 1. CRISPR screening primers | |
| Name | Sequences |
| CRISPR scrn1F | GAAATAGGGATCACGGCTGG |
| CRISPR scrn1R | CCGCAGCACTGGATTGTTAT |
| 1. *Mucin* sequencing primers | |
| Name | Sequences |
| Mucin­_HvHz_ex1_Fa | GCGATCGCTCTTCGTTCTAAC |
| Mucin­_HvHzex_2_Ra | GCCGAATACCACGATTCTGG |
| Mucin­_HvHz_ex2_Fb | CGGACTCCTTACCAGAATCG |
| Mucin­_HvHz_ex2_Rb | GTGGTCGTAGTTCTTCCAGG |
| Mucin­_HvHz_ex2_Fc | CTGAACAAACCCAACCACCA |
| Mucin­_HvHz_ex3_Rc | CAGCGACCGTCTAGAACCTT |
| Mucin­_HvHz_ex2_Fd | ACTGAACAAACCCAACCACC |
| Mucin­_Hv_ex3_Rd | GAGTCAACTGTCCTGATGCG |
| 4. CRISPR sgRNA | |
| Name | Sequences |
| D11-Guide1 | ATGGTCCCTCACCCAGTCAATGG |
| D11-Guide2 | CAGTATCGCTGTACTTATGATGG |
| D11-Guide3 | CATGGGAATAAAAGAACCCTCGG |

**
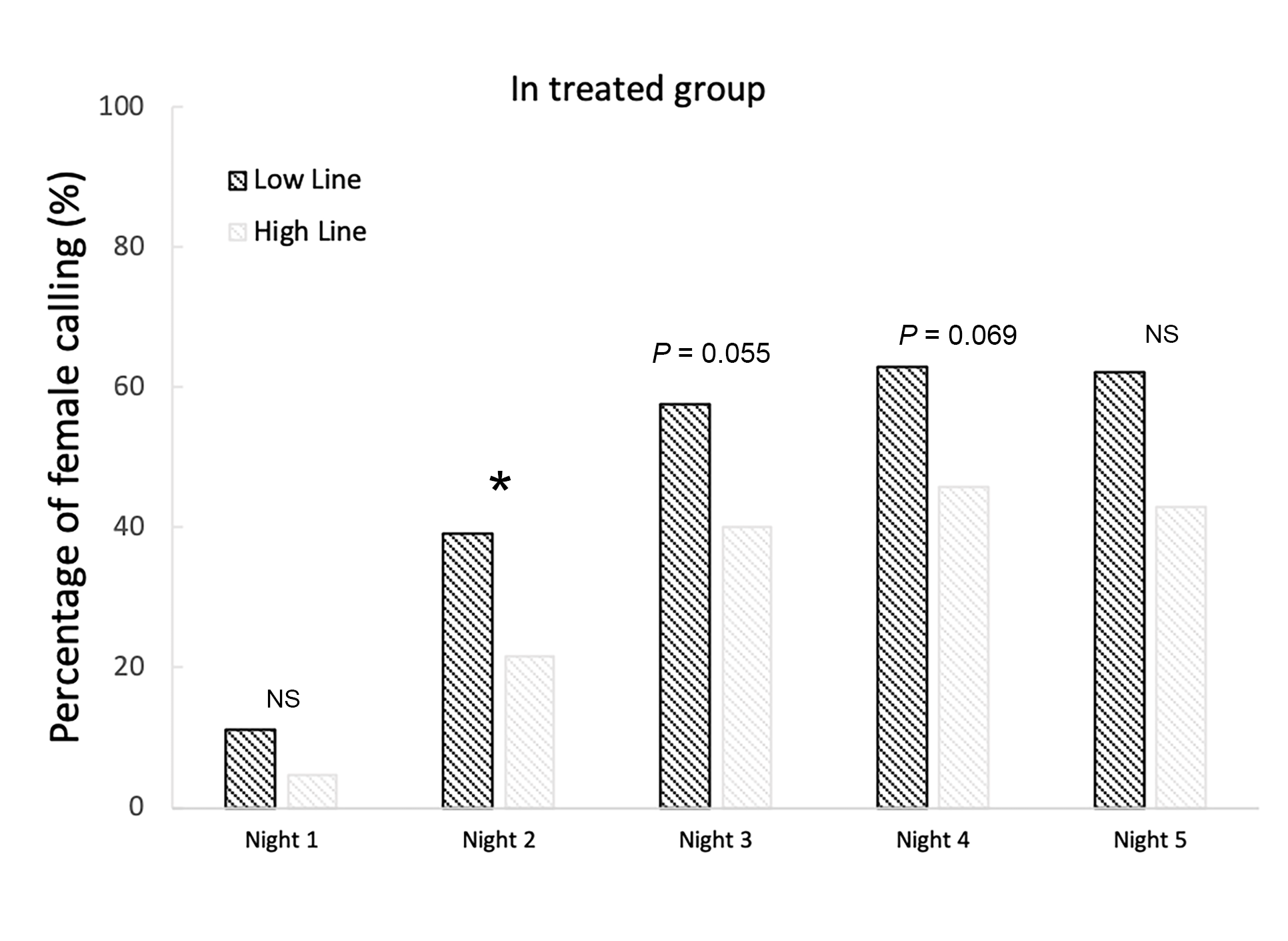
**

Fig. S1 Comparing the females calling behavior in the treated group between Low and High line throughout the scotophase over five consecutive nights. The number of females observed over five consecutive nights is given in Table S1. Significant differences are indicated by asterisks (*P* < 0.05); NS: not significant.
